# Fenofibrate Improved Interstitial Fibrosis of Renal Allograft through Inhibited Epithelial-Mesenchymal Transition Induced by Oxidative Stress

**DOI:** 10.1155/2019/8936856

**Published:** 2019-02-17

**Authors:** Yishu Wang, Lei Pang, Yanghe Zhang, Jiahui Lin, Honglan Zhou

**Affiliations:** ^1^Key Laboratory of Pathobiology, Ministry of Education, Jilin University, Changchun, 130021 Jilin, China; ^2^Department of Anesthesiology, The First Hospital of Jilin University, Changchun, 130021 Jilin, China; ^3^Department of Urology, The First Hospital of Jilin University, Changchun, 130021 Jilin, China

## Abstract

The best treatment for end-stage renal disease is renal transplantation. However, it is often difficult to maintain a renal allograft healthy for a long time following transplantation. Interstitial fibrosis and tubular atrophy (IF/TA) are significant histopathologic characteristics of a compromised renal allograft. There is no effective therapy to improve renal allograft function once IF/TA sets in. Although there are many underlying factors that can induce IF/TA, the pathogenesis of IF/TA has not been fully elucidated. It has been found that epithelial-mesenchymal transition (EMT) significantly contributes to the development of IF/TA. Oxidative stress is one of the main causes that induce EMT in renal allografts. In this study, we have used H_2_O_2_ to induce oxidative stress in renal tubular epithelial cells (NRK-52e) of rats. We also pretreated NRK-52e cells with an antioxidant (N-acetyl L-cysteine (NAC)) 1 h prior to the treatment with H_2_O_2_. Furthermore, we used fenofibrate (a peroxisome proliferator-activated receptor *α* agonist) to treat NRK-52e cells and a renal transplant rat model. Our results reveal that oxidative stress induces EMT in NRK-52e cells, and pretreatment with NAC can suppress EMT in these cells. Moreover, fenofibrate suppresses fibrosis by ameliorating oxidative stress-induced EMT in a rat model. Thus, fenofibrate may effectively prevent the development of fibrosis in renal allograft and improve the outcome.

## 1. Introduction

Renal transplantation is the best approach for the management of end-stage renal disease. However, it brings along with it the risk of graft failure or transplant rejection. With the use of novel and effective immunosuppressive agents, the incidence of transplant rejection has reduced substantially in recent years [1]. However, the long-term outcome of renal allograft has not improved much. Although the annual survival rate of renal transplant has reached more than 90%, there is a 4–5% loss of function in the renal graft per year. The 5-year survival rate of renal transplant is approximately 70%, whereas the 10-year survival rate is only around 50% [1]. The main cause of this sharp decline is the development of chronic allograft nephropathy (CAN) [2, 3]. In the new Banff 2007 scheme, the term chronic allograft nephropathy has been replaced by interstitial fibrosis/tubular atrophy (IF/TA) [4].

Clinical research has shown that IF/TA is a significant histopathologic characteristic of a compromised renal allograft [5] and IF/TA is associated with chronic renal allograft dysfunction [6]. Multiple studies have been conducted in the past decades to understand the pathogenesis of IF/TA. These studies have shown that a wide range of factors and mechanisms are involved in the progress of IF/TA. These factors can be classified into two main categories: immune and nonimmune. The immune factors are mostly immunosuppressive drug toxicity and antibody-mediated injury, while the nonimmune factors are vasoconstriction, oxidative stress, fibroblast activation, transforming growth factor beta- (TGF-) *β*1-mediated epithelial-mesenchymal transition (EMT), etc. [5].

EMT has been identified in many biological functions, including tissue regeneration, scars following injury, epithelial-derived tumor invasion, and metastases [7, 8]. Three types of EMT have been identified: (i) type I EMT which is involved in embryo development and organ formation, (ii) type II EMT which is involved in adult tissue repair and fibrosis, and (iii) type III EMT which refers to the phenotypic transformation of epithelial malignancies [9]. The results of sequential renal biopsies in a study performed 3 and 12 months posttransplant have demonstrated a close correlation between the development of IF/TA and expression of EMT markers [10]. Some researchers have compared the biopsies obtained from dysfunctional renal allografts with IF/TA and renal allografts with stable renal function. They have shown that the expression of epithelial markers (E-cadherin and cytokeratin) in tubular epithelial cells (TECs) of kidneys with IF/TA is significantly decreased and the distribution of the markers is altered as compared to the functional kidneys [11]. In the damaged and atrophic tubules, some TECs also expressed mesenchymal markers including vimentin, S100A4, and alpha-smooth muscle actin (*α*-SMA). These findings indicate that TEC damage is associated with EMT [11].

Oxidative stress is the main contributor to IF/TA [12]. Renal transplant recipients always manifest persistent oxidative stress during the early period posttransplant [13]. It was shown that oxidative stress increases in all the biopsy specimens with allograft dysfunction [14]. The oxidative stress occurring in the transplanted kidneys is mainly induced by immunosuppressive drugs involving cells of epithelial, endothelial, and mesenchymal origin. This also results in toxic effects and fibrosis of the renal allografts [15]. There is evidence that oxidative stress is involved in the pathogenesis of EMT in chronic IF/TA of renal allograft. One *in vitro* study using proximal tubular epithelial cells has demonstrated that reactive oxygen species (ROS) play an important role in TGF-*β*1-induced EMT through activation of mitogen-activated protein kinase (MAPK) and Smad pathways [16]. Another study using a rat model of kidney transplant revealed that EMT is involved in the development of IF/TA and it coexists with enhanced oxidative stress [17]. In this particular study, kidneys from Fisher 344 rats were transplanted in Lewis rats to develop a model of IF/TA. The presence of EMT was characterized by increased *α*-SMA and collagen I and III levels along with a decreased E-cadherin expression and increased superoxide anion and iNOS and eNOS levels depicting an increased oxidative stress. These observations suggest that EMT occurs concurrently with oxidative stress in IF/TA. Therefore, oxidative stress induced by immunosuppressive drugs may result in EMT and contribute to the development of IF/TA in a renal allograft [17].

Peroxisome proliferator-activated receptor *α* (PPAR*α*) is a member of the nuclear receptor superfamily, involved in the regulation of *β*-oxidation of fatty acids. PPAR*α* displays its biological functions by inducing the transcription of downstream target genes. PPAR*α* also has several antioxidant effects. A study has shown that fenofibrate (a PPAR*α* agonist) can significantly reduce the oxidative stress in kidneys of spontaneously hypertensive rats by reducing the activity of renal nicotinamide adenine dinucleotide phosphate (NADPH) oxidase, increasing the activity of Cu-Zn-superoxide dismutase, and decreasing the phosphorylation of p38 MAPK and c-Jun N-terminal kinase (JNK) signals [18]. Some authors have also shown that fenofibrate can restore the phenotypic change induced by the deficiency of LKB1 in TEC [19]. Another study has also revealed that fenofibrate markedly suppresses fibrosis in a mouse model of chronic kidney disease (CKD) by improving fatty acid oxidation [20]. However, it is unclear whether fenofibrate suppresses fibrosis by decreasing the oxidative stress levels in the transplant kidneys. Therefore, we hypothesize that fenofibrate treatment may suppress EMT by reducing oxidative stress levels in the renal tubular epithelial cells and may improve long-term outcome in renal transplant recipients.

## 2. Materials and Methods

### 2.1. Detection of Cell Viability

Collected NRK-52e cells were cultured in a DMEM. These cells were implanted into a 96-well plate and treated with 100 *μ*mol/L H_2_O_2_ for 0.5 h, 1 h, 1.5 h, 2 h, and 2.5 h. Subsequently, CCK8 was added and was incubated for 1 h. The optical density (OD) was recorded at 450 nm.

### 2.2. ROS and Malondialdehyde (MDA) Estimation

(i) ROS-dichloro-dihydro-fluorescein diacetate (DCFH-DA) method: 10 *μ*mol/L DCFH-DA was diluted by a serum-free DMEM and incubated at 37°C for 20 min in the dark and then again washed by the serum-free medium. Fluorescence intensity was detected by BioTek Epoch with an excitation wavelength at 488 nm and an emission wavelength at 525 nm. (ii) ROS-dihydroethidium (DHE) method: 5 *μ*mol/L of DHE was incubated at 37°C for 30 min in the dark. Subsequently, cells were incubated in 1X Hoechst 33342 for 30 min, and cells were washed in phosphate-buffered saline (PBS) thrice. Fluorescence microscopy was used to detect the fluorescence intensity. (iii) Malondialdehyde-thiobarbituric acid (MDA-TBA) method: collected medium with different treatments. By following the standard procedure, agential nos. 1, 2, 3, and 10 nmol/mL standard substance were added to the medium and incubated at 95°C for 40 min and then centrifuged at 4000 rpm for 10 min. The OD was detected using a microplate reader at 532 nm.

### 2.3. Western Blot Analysis

The collected cells were treated with H_2_O_2_, N-acetyl L-cysteine (NAC), and/or fenofibrate. These cells were lysed using RIPA lysis buffer containing a protease inhibitor cocktail and a phosphatase inhibitor. The sample concentrations were measured by the bicinchoninic acid (BCA) assay. The proteins were loaded and separated by 5%, 8%, or 10% sodium dodecyl sulfate (SDS) polyacrylamide gels, electrotransferred to PVDF membranes, and incubated for about 12 h at 4°C with the following antibodies: E-cadherin, N-cadherin, vimentin, Snail, cleaved caspase-3, S100A4, and GAPDH. Secondary antibodies (anti-rabbit or anti-mouse IgG horseradish peroxidase (HRP)) were diluted by tris-buffered saline and polysorbate 20 (TBST) and incubated for 1 h. The blots were detected by enhanced chemiluminescence.

### 2.4. Quantitative Real-Time PCR Analysis

Total RNA was prepared from rat renal tubular epithelial cells (NRK-52e) using an RNeasy Mini Kit. 500 ng of total RNA was reverse-transcribed using a cDNA archival kit. Quantitative real-time polymerase chain reaction was performed using an ABI 7300 real-time PCR System involving SYBR Green Master Mix, 3-step standard cycling conditions, and sequence-specific primers. The melting curve was examined to verify the amplification of a single product. For quantitative analysis, all the samples were normalized to ubiquitin C gene expression using the ΔΔCT value method.

### 2.5. Renal Transplant Model

Sprague Dawley rats were used as donors, and Wistar rats were used as recipients for transplantation of the left kidneys. Donor operation: 125 U/mL heparin saline was injected through the lumbar vein of the donor rat. Styptic clips were used to stop the blood supply. Perfusion was conducted through the aorta abdominalis, and the left renal vein was cut off at the same time. When perfusion finished, the left renal artery, vein, and ureter were ligated. The left kidney was separated and dipped into heparin saline at the temperature of 0~4°C. Recipient operation: the blood supply was stopped with styptic clips and the left kidney was separated. The renal artery, renal vein, and ureter of the donor were anastomosed to those of the recipient. The blood supply was restored after anastomotic surgery. Postoperative management: 5 mL saline was injected into the abdominal cavity after closing the abdomen. After renal transplantation, the Wistar rats were treated with penicillin for 3 days and cyclosporin A for 1 week. The rats were followed up for 8 weeks for the development of IF/TA.

### 2.6. Histological and Immunohistochemical Examination

Following 8 weeks posttransplant, the rats were euthanized and a section of both the kidneys was excised. The excised tissues were embedded in paraffin and optimal cutting temperature compound (OTC) to be made into paraffin sections and frozen sections (used into the ROS-DHE examination). Subsequently, the tissues were stained with different staining techniques such as hematoxylin and eosin (HE), periodic acid-Schiff (PAS), periodic acid-silver methenamine (PASM), Masson trichrome, and immunohistochemical examination.

#### 2.6.1. HE

The paraffin sections were dewaxed with a series of xylene and ethyl alcohol. Then, the sections were stained with hematoxylin (3 min) and eosin (10 min). Sections were dehydrated and then sealed with neutral resins.

#### 2.6.2. PAS

Dewaxed sections were stained with 1% periodic acid solution (10 min), then washed with water (10 min). These sections were dipped into Schiff solution (10 min) and washed with water (5 min). Then, these sections were stained with hematoxylin (3 min). Finally, sections were dehydrated with a series of ethyl alcohol and xylene and then sealed with neutral resins.

#### 2.6.3. PASM

Dewaxed sections were stained with 1% periodic acid solution (10 min), then washed with water (10 min). These sections were dipped into silver methenamine solution (1 h) and 3% sodium thiosulfate (20 s). Then, these sections were stained with hematoxylin for 3 min, dehydrated, and sealed.

#### 2.6.4. Masson

Dewaxed sections were stained with hematoxylin (3 min) and washed with water. Then, these sections were dipped into a ponceau-acid fuchsin solution (5 min), 2% glacial acetic acid (30 s), 1% phosphomolybdic acid (5 min), and aniline blue (5 min). Finally, sections were dehydrated and then sealed with neutral resins.

#### 2.6.5. Immunohistochemical Examination

Specific antibodies to Snail, S100A4, and vimentin were used, followed by an addition of horseradish peroxidase- (HRP-) conjugated secondary antibodies and 3,3-diaminobenzidine (DAB) for signal detection.

### 2.7. Statistical Analysis

The continuous variables were expressed as mean and standard deviation. One-way analysis of variance was performed followed by the least significant difference test. A *p* value of < 0.05 was considered statistically significant.

## 3. Results

### 3.1. Oxidative Stress Induces EMT in Rat Renal Tubular Epithelial Cells

**Figure 1:**
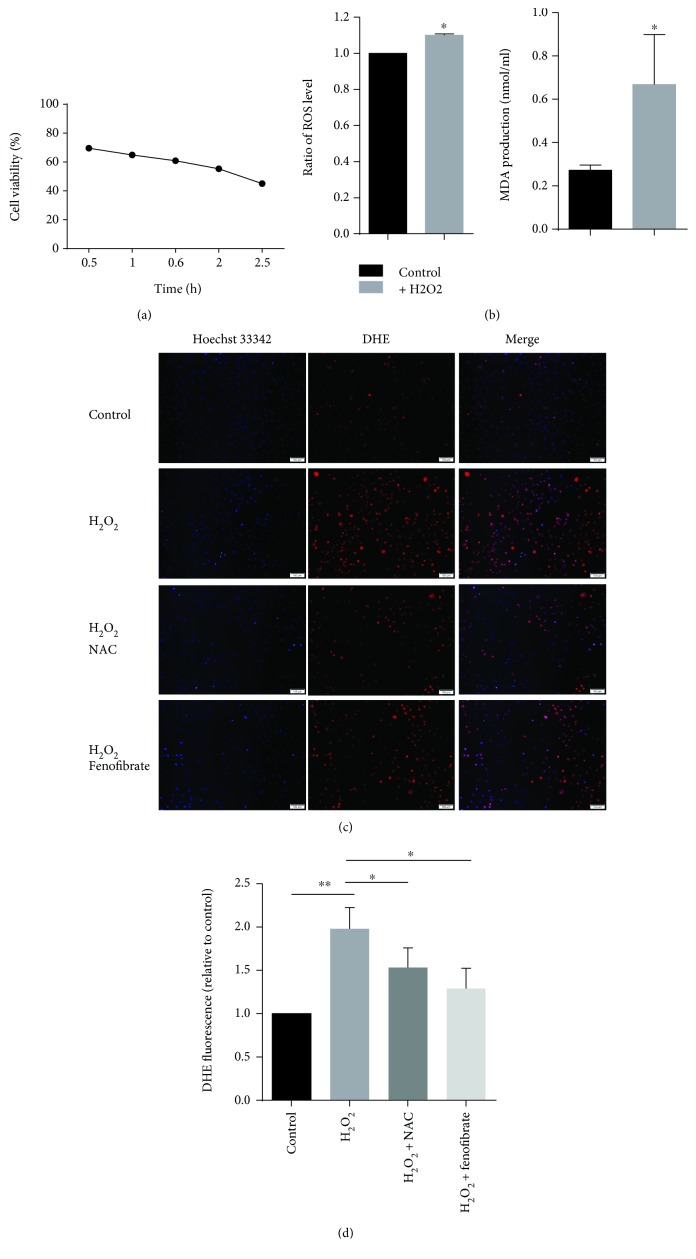
H_2_O_2_ treatment induced oxidative stress in rat renal tubular epithelial cells. (a) NRK-52e cells were treated with 100 *μ*mol/L H_2_O_2_ for 0.5 h, 1 h, 1.5 h, 2 h, and 2.5 h, and cell viability was detected with CCK8. (b) NRK-52e cells were treated with 100 *μ*mol/L H_2_O_2_ for 1 h and 2 h; relative ROS levels were detected with the DCFH-DA method. Cells were treated with H_2_O_2_, and the medium of the control group was changed at the same time, and then, the medium of cells was collected with or without H_2_O_2_ treatment 2 h later, and MDA levels were detected. (c) NRK-52e cells were pretreated with 1 mmol/L NAC or 1 *μ*mol/L fenofibrate for 1 h, followed by treatment with 100 *μ*mol/L H_2_O_2_ for 2 h. DHE and Hoechst 33342 were incubated for 30 min, and a fluorescence microscope was used to observe the fluorescence intensity (red, DHE, exposure time (1.3 s); blue, Hoechst 33342, exposure time (70 ms)). (d) Quantitative analysis was conducted with Image-Pro Plus. ^∗^*p* < 0.05, ^∗∗^*p* < 0.01.

To determine whether oxidative stress is associated with EMT, we treated the rat renal tubular epithelial cell line (NRK-52e cells) with 100 *μ*mol/L H_2_O_2_ and studied the phenotypic changes. With longer exposure to H_2_O_2_, the cell viability decreased (Figure 1(a)), and there was a significant difference among each group. H_2_O_2_ treatment for 2 h increased the ROS (DCFH-DA method) and MDA levels in the cells (Figure 1(b)). The ROS level detected using a fluorescence microscope (Figures 1(c) and 1(d)) also demonstrated that H_2_O_2_ treatment induced oxidative stress.

**Figure 2:**
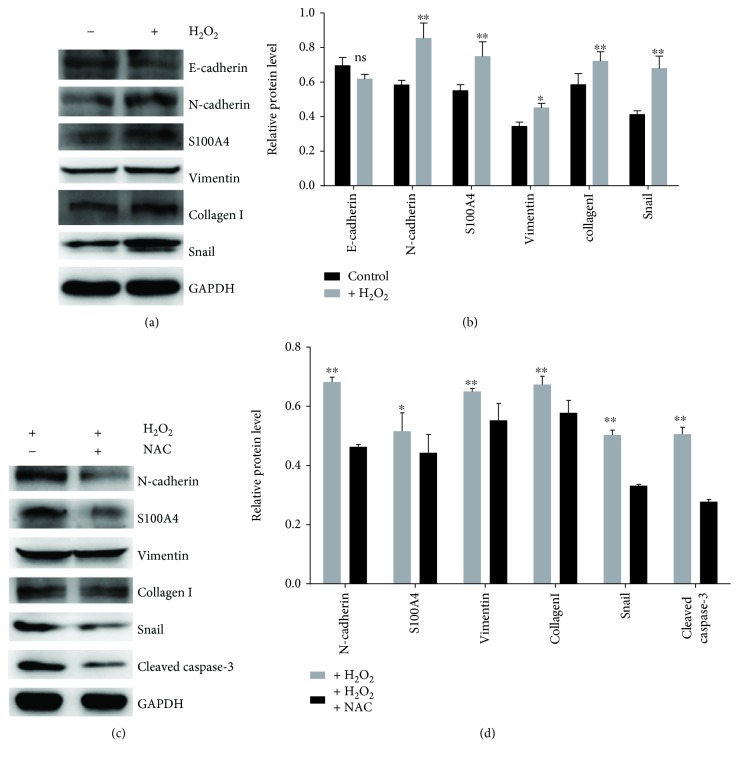
Oxidative stress-induced EMT in rat renal tubular epithelial cells. (a) NRK-52e cells were treated with 100 *μ*mol/L H_2_O_2_ for 2 h and cultured for 24 h, and then Western blot was conducted to detect protein expression. (b) Quantitative analyses were conducted with ImageJ. (c) The cells were pretreated with 1 mM NAC, then treated with 100 *μ*mol/L H_2_O_2_ for 2 h. The expression of N-cadherin, vimentin, S100A4, collagen I, Snail, and cleaved caspase-3 was detected by Western blot. (d) Quantitative analyses were conducted with ImageJ. ^∗^*p* < 0.05, ^∗∗^*p* < 0.01.

To define whether oxidative stress induces EMT in NRK-52e cells, we conducted Western blots to detect EMT-related markers. We found that the expression of N-cadherin, S100A4, vimentin, collagen I, and Snail appears to increase in the cells treated with H_2_O_2_ (Figures 2(a) and 2(b)).

To further clarify that EMT in NRK-52e cells was induced by oxidative stress, we pretreated the NRK-52e cells with an antioxidant (NAC) for 1 h, followed by a treatment with 100 *μ*mol/L H_2_O_2_ for 2 h. Our results revealed that NAC pretreatment significantly decreased the ROS level (Figures 1(c) and 1(d)) and reversed the phenotypic changes as evidenced by the decreased expression of N-cadherin, S100A4, vimentin, collagen I, and Snail (Figures 2(c) and 2(d)). As oxidative stress induces cell apoptosis, we also tested the expression of cleaved caspase-3, and our results demonstrated that NAC decreased the cleaved caspase-3 expression (Figures 2(c) and 2(d)). These results indicated that enhanced oxidative stress induced EMT in renal tubular epithelial cells.

### 3.2. Fenofibrate Reduced EMT *via* Suppression of Oxidative Stress in Rat Tubular Epithelial Cells

**Figure 3:**
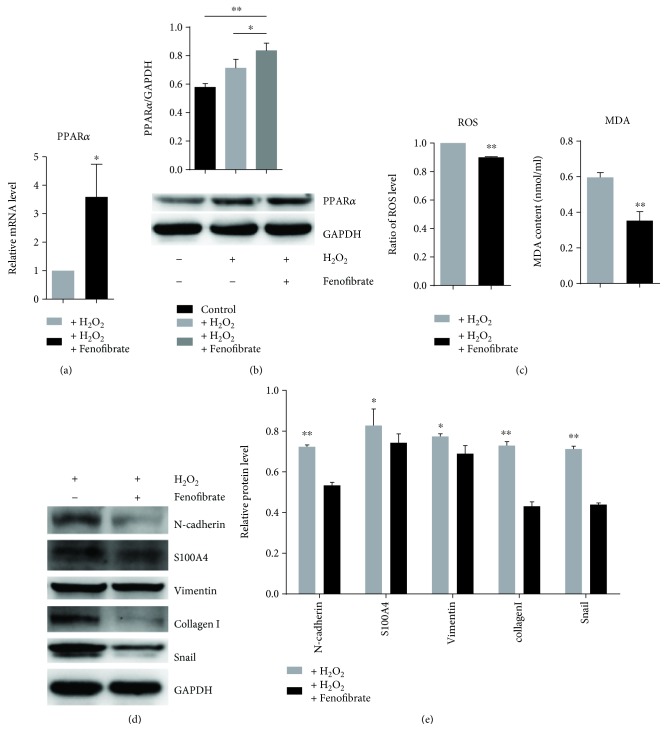
Fenofibrate recovered phenotypic changes induced by oxidative stress. (a, b) PPAR*α* expression level assessed *via* quantitative RT-PCR and Western blot analysis in NRK-52e cells treated with H_2_O_2_ for 2 h and then with fenofibrate for 24 h. (c) Relative ROS and MDA levels after treatment with fenofibrate and H_2_O_2_. (d, e) Western blot and quantitative analysis for the expression of N-cadherin, S100A4, vimentin, collagen I, and Snail with ImageJ. ^∗^*p* < 0.05, ^∗∗^*p* < 0.01.

We studied the effect of fenofibrate on oxidative stress. We treated the NRK-52e cells with H_2_O_2_ and/or fenofibrate and detected the oxidative stress level. When treated with fenofibrate, the mRNA and protein levels of PPAR*α* were increased (Figures 3(a) and 3(b)), and the ROS and MDA levels were significantly reduced (Figures 1(c), 1(d), and 3(c)). Further, we tested whether fenofibrate can reverse the phenotypic change of the cells induced by oxidative stress. We conducted Western blots to detect the expression of N-cadherin, S100A4, vimentin, collagen I, and Snail, and all these were found to be reduced (Figures 3(d) and 3(e)). These results indicate that fenofibrate can suppress oxidative stress-induced EMT.

### 3.3. Fenofibrate Suppresses Fibrosis of Transplant Kidneys in Rats

**Figure 4:**
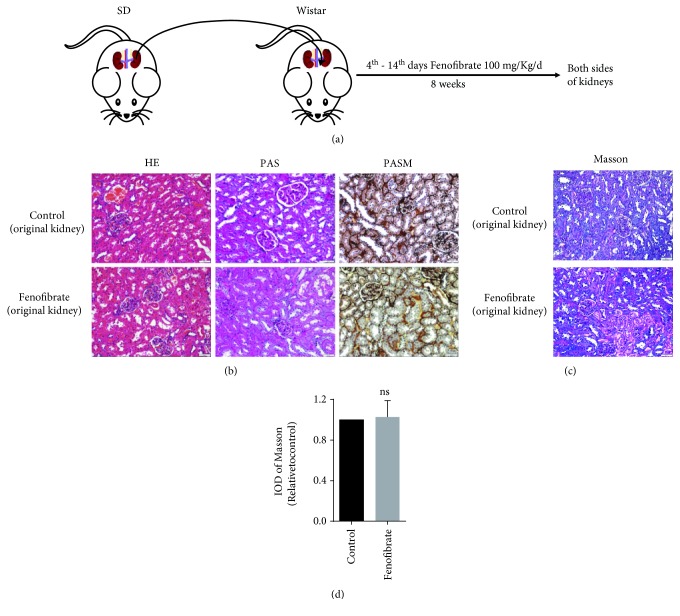
Renal transplantation models were established. (a) Wistar rats were used to make unilateral kidney transplant models with kidneys obtained from Sprague Dawley rats. The experimental group was pretreated with 100 mg/kg/d of fenofibrate for 10 d, and the control group was treated with the same solvent after transplant. (b) HE, PAS, and PASM histochemical stains for the original kidneys of the 2 groups of rats. (c) Masson histochemical stains for the original kidneys of the 2 groups of rats. (d) Quantitative analyses were conducted with Image-Pro Plus. IOD: integrated optical density.

Our results indicate that fenofibrate suppressed EMT by decreasing oxidative stress level in an *in vitro* model. Based on these results, we hypothesize that fenofibrate may inhibit interstitial fibrosis in a renal allograft. To further validate our conjecture, we used Sprague Dawley rats as donors and Wistar rats as recipients in the renal transplantation model (Figure 4(a)). One group was treated with fenofibrate for 10 d and another group was treated with solvent as a control posttransplantation. The rats were euthanized 8 weeks after transplantation, and the kidneys were excised. We used these kidney tissues for histochemical staining, including HE, Masson, PAS, and PASM, to define the therapeutic effect of fenofibrate for fibrosis of the transplanted kidneys. Our results revealed that the nontransplanted kidneys had no significant changes. The structure of renal glomerulus and renal tubules was normal, the capillary loops of the renal glomerulus were open, and there was no or little inflammatory cell infiltration in the renal mesenchyme (Figure 4(b)). There is also no significant difference of collagen fiber deposition in the original kidneys of the two groups (Figures 4(c) and 4(d)).

**Figure 5:**
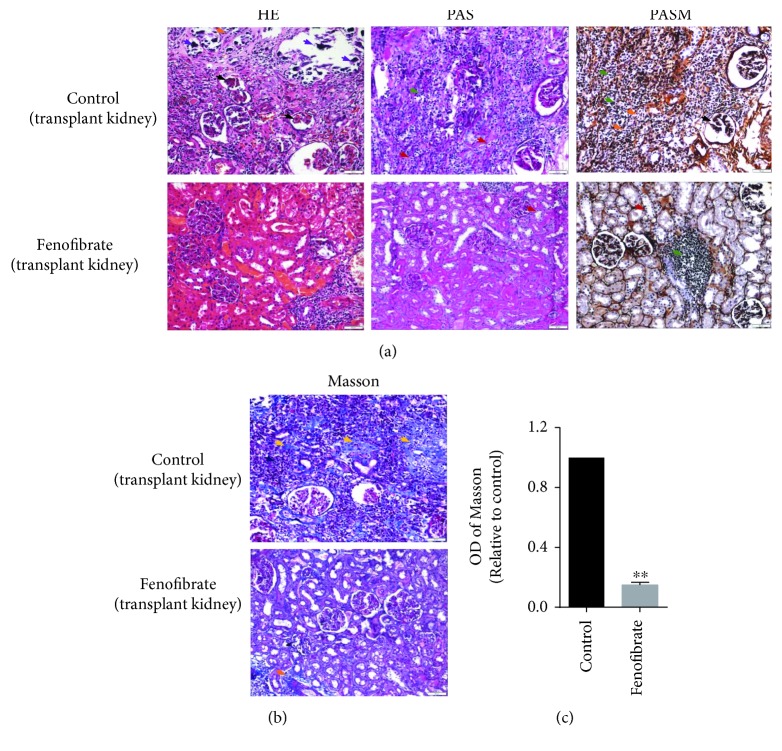
Fenofibrate improved fibrosis of renal allografts. (a, b) HE, PAS, PASM, and Masson histochemical stains for transplanted kidneys of 2 groups of rats (black arrow, renal glomerular shrinkage and capillary loop occlusion; red arrow, tubulitis; orange arrow, renal tubular atrophy; green arrow, inflammatory cell infiltrations; purple arrow, calcification; and yellow arrow, collagen fiber deposition). (c) Quantitative analyses were conducted with Image-Pro Plus. IOD: integrated optical density. ^∗∗^*p* < 0.01.

**Figure 6:**
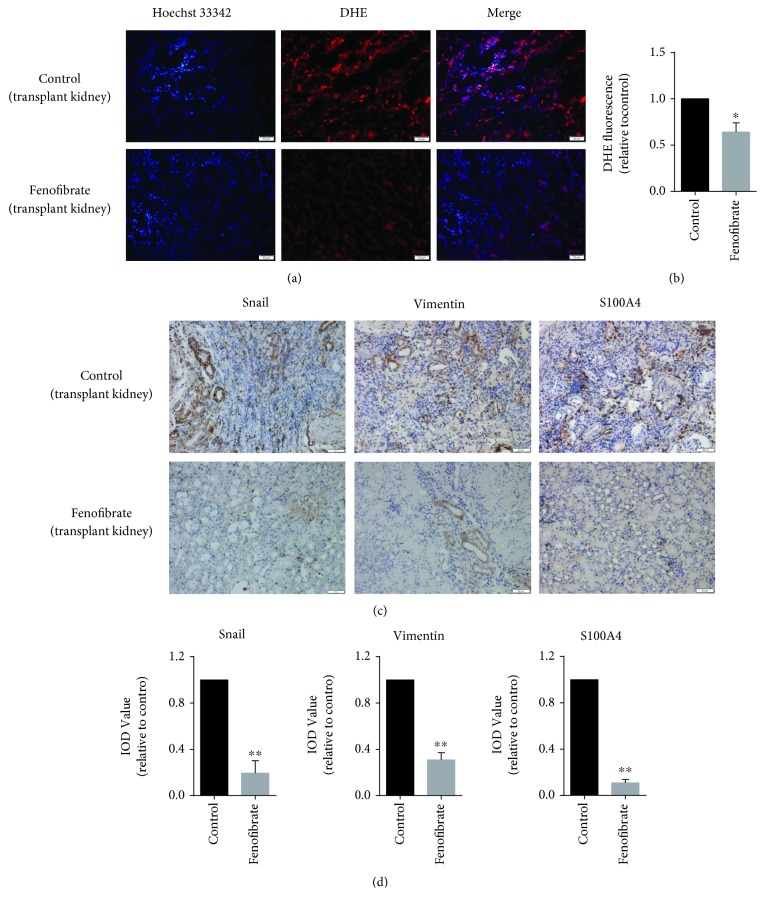
Fenofibrate suppresses EMT of renal allografts. (a) ROS detection with frozen sections. (red, DHE, exposure time (1.5 s); blue, Hoechst 33342, exposure time (50 ms). (b) Quantitative analyses were conducted with Image-Pro Plus. (c, d) Immunohistochemical findings and quantitative analysis for Snail, S100A4, and vimentin expressions. ^∗^*p* < 0.05, ^∗∗^*p* < 0.01.

The transplanted kidneys derived from the control group demonstrated tubulitis, renal glomerular shrinkage, renal tubular atrophy, capillary loop occlusion, a large number of inflammatory cell infiltrations, collagen fiber deposition, and calcification (Figures 5(a), 5(b), and 5(c)). Fenofibrate significantly improved the fibrotic changes in renal allografts. In the fenofibrate-treated groups, the structure of the glomerulus and tubules was relatively normal, the capillary loops of the renal glomerulus were open, and there were only minor inflammatory cell infiltrations, tubulitis, and collagen fiber depositions (Figures 5(a), 5(b), and 5(c)). In addition, fenofibrate treatment of the transplanted kidney significantly decreased the ROS level (Figures 6(a) and 6(b)). Immunohistochemical findings also showed that Snail, S100A4, and vimentin were positively expressed in the control group, and the fenofibrate treatment decreased the expression of these proteins (Figures 6(c) and 6(d)). These results further suggested that fenofibrate suppressed EMT in renal allografts. Taken together, our results exhibited that fenofibrate markedly improved chronic fibrosis of the renal allograft by suppressing EMT.

## 4. Discussion

**Figure 7:**
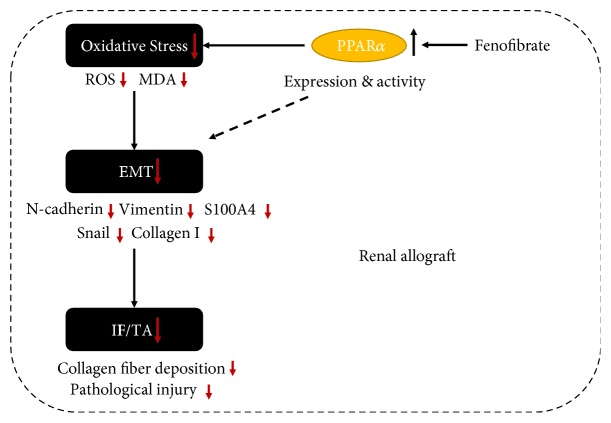
Model. Fenofibrate improved interstitial fibrosis of the renal allograft through inhibiting EMT induced by oxidative stress. Fenofibrate increased the expression and activity of PPAR*α* and suppressed EMT induced by oxidative stress (imaginary line showed other mechanisms, such as the fatty acid oxidation pathway), thereby reducing collagen fiber deposition and improving pathological injury of the renal allograft. Fenofibrate effectively delays the progress of IF/TA.

In our study, we defined that H_2_O_2_ increased the oxidative stress level in NRK-52e cells. EMT was induced by strengthened oxidative stress, and the fenofibrate treatment suppressed EMT induced by oxidative stress *in vitro*. To further determine the therapeutic effect of fenofibrate for IF/TA in the renal allograft, we established rat renal transplantation models and treated the models with fenofibrate. Results *in vivo* showed that fenofibrate significantly improved fibrosis and pathological injury in the renal allograft; that is, the structure of the glomerulus and tubules was relatively normal, the capillary loops of the renal glomerulus were open, and there were only minor inflammatory cell infiltrations, tubulitis, and collagen fiber depositions. The fenofibrate treatment also decreased the ROS level and suppressed EMT in the renal allograft (Figure 7).

Oxidative stress often leads to injury and fibrosis in a renal allograft following transplantation. The balance between the production of ROS and the defense against ROS defines the degree of oxidative stress *in vivo*. ROS act as signal and regulatory molecules to participate in cell proliferation, differentiation, and apoptosis [21–23]. A prooxidant microenvironment can alter and denature carbohydrates, nucleic acids, proteins, and lipids resulting in cell toxicity. There are multiple reports revealing the deleterious effects of oxidative stress resulting in different pathophysiologic states, such as neoplasm [24], aging [25, 26], cardiovascular diseases [27–31], and CKD [32–35].

Transplant surgery certainly leads to perioperative acute kidney injury (AKI) from ischemia-reperfusion (IR). It is to be noted here that AKI itself induces ROS generation and cell apoptosis, which contributes to the process of interstitial fibrosis [36]. However, AKI occurred in an early stage after the operation of renal transplant; IF/TA is a long-term and tardy course of the renal allograft. There are also multiple other factors leading to a higher oxidative stress, and one of which is the use of immunosuppressant drugs. All the renal transplant recipients experience a higher oxidative stress as evidenced by elevated specific biomarkers [12, 37–42]. As discussed, oxidative stress is a common mechanism of injury in chronic allograft IF/TA which leads to EMT [12]. From this study, we have demonstrated that increased oxidative stress in rat renal tubular epithelial cells induces EMT.

PPAR*α* is a transcription factor which is widely expressed in multiple organs like the liver, heart, and kidney. Activation of PPAR*α* results in the protection against ischemia-reperfusion injury induced by myocardial ischemia [43]. The activation of PPAR*α* protects myocardial cells by increased expression and activation of superoxide dismutase (SOD1, SOD2) and catalase and suppresses the generation of ROS in myocardial ischemia [43]. Fenofibrate (a PPAR*α* agonist) treatment for 18 weeks had shown to suppress the expression of P47^phox^ (a subunit of NADPH oxidase) and increase the activation and expression of Cu/Zn-SOD in a spontaneously hypertensive rat model [44]. Fenofibrate also exerted protective effects in hypertensive nephropathy and improved renal tubule interstitial fibrosis, glomerular sclerosis, and inflammatory cell infiltration [18]. The activation of PPAR*α* exhibits protection by suppressing oxidative stress in multiple animal models including alcoholic liver disease [45–47], diabetic retinopathy [48], and Parkinson's disease [49].

In this study, fenofibrate decreased the oxidative stress level and suppressed EMT in NRK-52e cells. PPAR*α* displays its biological functions by transcription regulation and activates multiple endogenous antioxidants, including SOD1, SOD2, and catalase [50]. PPAR*α* is an important transcription factor involved in crucial metabolic pathways like *β*-oxidation of fatty acids. Multiple studies have demonstrated that increased fatty acid oxidation improves interstitial fibrosis in CKD [19, 20]. Fenofibrate may also suppress EMT by the fatty acid oxidation pathway in the transplanted kidneys. In our study, fenofibrate treatment after kidney transplant decreased the injury of the renal allograft and improved the fibrosis state. However, the mechanism of suppression of EMT by fenofibrate may not be solely explained by the decreased oxidative stress, and it may also involve fatty acid oxidation pathways. Further mechanistic studies are warranted to investigate this role of fenofibrate in suppressing EMT in renal allografts.

In conclusion, fenofibrate suppressed EMT induced by oxidative stress *in vitro* and *in vivo*. The oxidative stress level was increased in the renal allograft of rats, which is a factor to contribute to IF/TA. Fenofibrate treatment decreased the ROS level of the renal allograft. The EMT progress was also suppressed by fenofibrate. Taken together, fenofibrate may delay the progress of IF/TA in the renal allograft through suppressing EMT induced by oxidative stress.

## Data Availability

The data used to support the findings of this study are available from the corresponding author upon request.
